# Lipoicmethylenedioxyphenol Reduces Experimental Atherosclerosis through Activation of Nrf2 Signaling

**DOI:** 10.1371/journal.pone.0148305

**Published:** 2016-02-09

**Authors:** Zhekang Ying, Minjie Chen, Xiaoyun Xie, Xiaoke Wang, Nisharahmed Kherada, Rajagopal Desikan, Georgeta Mihai, Patrick Burns, Qinghua Sun, Sanjay Rajagopalan

**Affiliations:** 1 Department of Cardiology, East Hospital, Tongji University School of Medicine, Shanghai, 200120, PR China; 2 Department of Medicine Cardiology Division, University of Maryland School of Medicine, Baltimore, Maryland, 21201, United States of America; 3 Davis Heart & Lung Research Institute, Colleges of Medicine, The Ohio State University, Columbus, Ohio, United States of America; 4 Division of Geriatric Medicine, Tongji Hospital, Tongji University School of Medicine, Shanghai, PR China; 5 InVasc Therapeutics, Tucker, Georgia, United States of America; William Harvey Research Institute, Barts and The London School of Medicine and Dentistry, Queen Mary University of London, UNITED KINGDOM

## Abstract

**Objective:**

Oxidative stress is implicated in the pathogenesis of atherosclerosis, and Nrf2 is the transcriptional factor central in cellular antioxidant responses. In the present study, we investigate the effect of a dihydrolipoic acid derivative lipoicmethylenedioxyphenol (LMDP) on the progression of atherosclerosis and test whether its effect on atherosclerosis is mediated by Nrf2.

**Methods and Results:**

Both magnetic resonance imaging (MRI) scanning and en face analysis reveal that 14 weeks of treatment with LMDP markedly reduced atherosclerotic burden in a rabbit balloon vascular injury model. Myograph analyses show decreased aortic contractile response to phenylephrine and increased aortic response to acetylcholine and insulin in LMDP-treated animals, suggesting that LMDP inhibits atherosclerosis through improving vascular function. A role of Nrf2 signaling in mediating the amelioration of vascular function by LMDP was supported by increased Nrf2 translocation into nuclear and increased expression of Nrf2 target genes. Furthermore, chemotaxis analysis with Boydem chamber shows that leukocytes isolated from LMDP-treated rabbits had reduced chemotaxis, and knock-down of Nrf2 significantly reduced the effect of LMDP on the chemotaxis of mouse macrophages.

**Conclusion:**

Our results support that LMDP has an anti-atherosclerotic effect likely through activation of Nrf2 signaling and subsequent inhibition of macrophage chemotaxis.

## Introduction

Atherosclerosis is the principal cause of coronary artery disease, cerebrovascular accidents and gangrene of the extremities. There is a consensus that atherosclerotic lesions result from an excessive, inflammatory-fibro-proliferative response to various forms of insult to the endothelium and smooth muscle of the arterial wall. As a critical component of inflammatory responses, oxidative stress has been shown to mediate vascular damage in several classic risk factors for atherosclerosis, such as hypertension [1], dyslipidemia [2], and obesity [2]. Furthermore, there is evidence that oxidative stress plays a role in human atherosclerosis [3], and the administration of antioxidants reduces experimental atherosclerosis [4]. However, some recent clinical trials demonstrate disconnects between the predicted benefit and the clinical results of antioxidant therapy [4], indicating that there are still gaps in our knowledge regarding the role of oxidative stress in intravascular pathophysiology and the selection of effective antioxidant therapy.

Mechanisms to reduce the risk of oxidative stress have been evolved in living cells, such as the antioxidant enzyme system comprised of superoxide dismutase, catalase, and glutathione peroxidase. The transcriptional factor nuclear factor-(erythroid-derived 2) like 2 factor (Nrf2) is central in the expression of these antioxidant enzymes. Its activation has been shown to protect endothelial cells from oxidant injury [5], suppresses smooth muscle cell proliferation [6], and reduce arterial pro-inflammatory state [7], suggesting that Nrf2 may have an anti-atherosclerotic function. However, whole-body deletion of Nrf2 unexpectedly was shown to promote the progression of atherosclerosis [8], probably subsequent to dysfunction of plasma lipoproteins [9] and cholesterol crystal-induced inflammasome activation [10]. In contrast, myeloid deletion of Nrf2 markedly reduces atherosclerosis [11], suggesting that the role of Nrf2 in atherosclerosis is cell type-dependent and further investigations are warranted. Notably, a recent study showed that dietary Nrf2 activators inhibit atherogenic processes [12], supporting that Nrf2 pathway is an anti-atherosclerotic target.

Along with others [13–15], we previously demonstrated that lipoic acid, a co-enzyme with anti-oxidant and anti-inflammatory effects, has a potent anti-atherosclerotic effect [16]. Interestingly, several studies have suggested that the anti-oxidant and anti-inflammatory effects of lipoic acid may be mediated by Nrf2 activation [17–19]. However, there is no study investigating the role of Nrf2 activation in the anti-atherosclerotic effect of lipoic acid. In addition, we recently revealed that a modified methylenedioxyphenol derivative markedly inhibits the progression of atherosclerosis through inhibition of inflammatory response [20]. In the present study, we synthesized a novel compound lipoicmethylenedioxyphenol (LMDP) that has the moieties of both lipoic acid and methylenedioxyphenol, tested its efficacy on atherosclerotic progression in a balloon injury-induced rabbit atherosclerosis, and investigated the role of Nrf2 activation in its anti-atherosclerotic effect with both *in vivo* and *in vitro* systems.

## Materials and Methods

### Animals

12 male New Zealand White rabbits were bought from Charles River, and fed with high fat diet (HFD) (Harlan Teklad rabbit diet with 0.5% cholesterol, TD 87251) throughout the study. After 4 weeks of high fat diet alone rabbits were randomized into control (n = 6) or LMDP (100 mg/kg/day; n = 6). LMDP was provided by mixing it with HFD. 2 weeks after LMDP administration, rabbits were subjected to aortic balloon denudation surgeries followed by other 12 weeks of LMDP treatment. For survival surgery, rabbits were anesthetized with isoflurane in oxygen (1.5–2.5%). Animal were euthanized with sodium pentobarbital (100 mg/kg, IP) followed by CO_2_. All procedures of animal studies were approved by the Institutional Animal Care and Use Committee (IACUC) of the Ohio State University (Protocol #2009A0195-R1).

### Experimental Balloon Vascular Injury

Atheroma was induced in the abdominal aorta by the combination of HFD and balloon-induced intimae injury. Rabbits were anesthetized with isoflurane in oxygen (1.5–2.5%). The cardiovascular system was monitored by a pulse oximeter applied to the rabbit cheek. Both left and right medial thigh and peritoneal region were aseptically prepared for the surgery. 3–4 cm incision was made in the area of the femoral triangle and the femoral artery was exposed, ligated distally with 1.5 metric braided lactomer (“Polysorb” Syneture, USA) and 0.2 ml of 5% solution of papaverine was applied to the vessel topically to provide enhanced vasodilatation. A small hole was made into the artery using micro scissors through which a 4F introducer (Avanti®+, Cordis) was advanced. After placement, the introducer was flushed with heparinized saline. A guide wire (0.014” diameter and 190 cm long) was advanced through the introducer into the femoral artery and abdominal aorta up to the level of diaphragm with the use of fluoroscope unit. A balloon dilation catheter NCM 15/3.5 model (NC Monorail™, Boston Scientific, Inc) was then threaded overtop of the guide wire and advanced to the desired location within the abdominal aorta. The balloon was inflated using saline mixed contrast (Omnipaque/iohexol 350mg/ml). The contrast agent within the balloon allows for visualization of inflation. The balloon was inflated up to its nominal compliance using 8.0 ATM pressure to provide adequate resistance and moved back and forth up to aortic bifurcation to induce denudation injury to the whole abdominal aorta. After successful inflation, the balloon, guide wire and introducer were removed. Femoral artery was ligated (there is enough collateral blood supply to the leg that this does not typically cause any adverse effects). The muscle layer was closed using 3–0 polypropylene interrupted sutures and the skin was re-apposed using intradermal 3–0 polypropylene continuous sutures.

### Plasma Lipids

Basic lipid profile (Cholesterol, Triglycerides, direct HDL, and direct LDL) was done at Cardiovascular Specialty Laboratories, Inc. Atlanta. GA. 1 ml of blood was taken into K EDTA tubes at the start of high cholesterol diet and again at the time of sacrifice.

### Magnetic Resonance Imaging (MRI)

MRI experiments were conducted at two time points, 1 week and 12 weeks post balloon denudation procedure. Preceding MRI acquisitions, rabbits were anesthetized with xylazine (2mg/kg) and ketamine (40 mg/kg). Pre and post gadolinium dark blood acquisitions using a double inversion T1-weighted gradient echo turbo FLASH sequence were obtained using a 1.5T 32-channel whole body MR system (MAGNETOM Avanto, Siemens, Germany). For imaging, rabbits were placed in prone position and were wrapped in a flexible 6-element phase array body coil. Thirty 4mm thick transversal slices (4.6 mm gap between slices), perpendicular on the aorta spanning approximately from the iliac bifurcation to the superior pole of the topmost kidney, were acquired with: TR/TE = 260/5ms, 312x312 μm in-plane resolution, bandwidth 120 kHz, three signal averages and a total scan time of 12:29 minutes. Following the pre-contrast acquisition 7–10 ml solution of 0.1mmol/Kg Magnevist ((Bayer HealthCare Pharmaceuticals Inc. Wayne, NJ) was injected through the catheter and flushed with 2 ml of saline. A repeat of the pre-contrast dark blood scan was started approximately 6:30 minutes post contrast administration. After the post-contrast scan rabbits were allowed to recover.

### MRI Data Analysis

MRI images were analyzed using a Siemens Leonardo Workstation (Siemens Healthcare Inc. Germany). To assess the rates of atherosclerosis plaque progression, the aortic wall was identified in the pre-contrast images. Regions of interest (ROI) were free hand drawn around the inner and outer vessel wall border in 10 to 22 images for each animal. Vessel wall area was obtained in each slice by subtracting the lumen area from the area encapsulated by the outer border of the vessel wall. A total vessel wall volume for each animal was obtained by multiplying the slice thickness (4.6mm) with the total vessel wall area (in mm). To account for the variable number of slices, for each rabbit a normalized aortic wall volume was calculated.

### Histological Analysis

After euthanasia, aortas were carefully removed from the arch to the iliac bifurcation, along with the heart and both kidneys, and placed into PBS. The heart, kidneys and iliac bifurcation served as important landmarks to orientate the aorta including the segment of abdominal aorta imaged by MRI. MRI images and histological sections were matched using a similar method to that validated by Worthley et al [21]. The region of abdominal aorta imaged by MRI as described above was excised, cut into 2 mm long segments of and embedded in Optimal Cutting Temperature (OCT) compound (Tissue-Tek, Sakura Finetek USA Inc, Torrance, Calif) and then frozen in liquid nitrogen which were then after ready for histology. Individual MRI slices were matched with histological blocks of aorta by measuring the distance from the renal arteries. The 2 mm long histological segments, matching the corresponding MRI images were serially sectioned, care being taken to monitor the orientation of the sections throughout. At least three sections from each segment were image-analyzed as described below. OCT sections (5 μm) were stained with haematoxylin and eosin and oil red O stain and also used for immunohistochemistry. Each image was digitized with a digital camera and analyzed under a research microscope (Zeiss Axioskop with Spot I digital camera, Jena, Germany) with National Institutes of Health (NIH) Image software version 1.61 (Wayne Rasband, NIH, http://rsb.info.nih.gov/nih-image). All analyses were performed blindly without knowledge of the origin of the samples.

To measure spontaneous atherosclerosis, segments of descending thoracic aorta were embedded in Optimal Cutting Temperature compound (Tissue-Tek, Sakura Finetek USA Inc, Torrance, Calif) and frozen on dry ice. En face sections were then prepared. To analyze atherosclerotic burden, 8–12 sections (4 μm thick) were collected at intervals of 20 μm. After H&E staining and Oil-red O staining, each section was analyzed in a blinded manner after digitizing the images. The images were analyzed under a research microscope (Zeiss Axioskop with Spot I digital camera, Jena, Germany) with National Institutes of Health (NIH) Image software version 1.61 (Wayne Rasband, NIH, http://rsb.info.nih.gov/nih-image). Results were normalized by the wall of aorta.

### Myograph Studies

The thoracic aortas were collected for the myograph studies. 2 mm thoracic aortic rings were suspended in individual organ chambers filled with physiological salt solution buffer (sodium chloride, 130 mEq/L; potassium chloride, 4.7 mEq/L; calcium dichloride, 1.6 mEq/L; magnesium sulfate, 1.17 mEq/L; potassium diphosphate, 1.18 mEq/L; sodium bicarbonate, 14.9 mEq/L; EDTA, 0.026 mEq/L; and glucose, 99.1 mg/dL [5.5 mmol/L]; pH, 7.4), aerated continuously with 5% carbon dioxide in oxygen at 37°C. Vessels were allowed to equilibrate for at least 1 hour at a resting tension of 30 mN before being subjected to graded doses of agonists. The vasoconstrictor agonists included phenylephrine (PE), endothelin-1 (ET-1), or angiotensin II. Responses were expressed as a percentage of the peak response to 120 mEq/L of potassium chloride. The vessels subjected to PE were washed thoroughly and allowed to equilibrate for 1 hour before beginning experiments with acetylcholine or SNP. After a stable contraction plateau was reached with PE (0.1 μM), the rings were exposed to graded doses of the endothelium-dependent agonist acetylcholine or the endothelium-independent agonist SNP. Results were expressed as a percentage of pre-contraction by PE (0.1 μM). The rings exposed to acetylcholine were thoroughly washed and allowed to equilibrate for 1 hour. After a stable contraction plateau was reached with PE (0.1 μM), insulin was then added in an accumulative manner. Results were expressed as a percentage of pre-contraction by PE (0.1 μM).

### Immunohistochemistry

**S**egments of thoracic aorta were embedded in Optimal Cutting Temperature compound (Tissue-Tek, Sakura Finetek USA Inc, Torrance, Calif) and then frozen in liquid nitrogen. The atherosclerotic burden was analyzed as previously shown.[16] Briefly, 4 successive sections were collected on the same slide, and at least 10 sections from 3 consecutive slides per area per mouse (sinus and thoracic aorta) were examined. Each image was digitized with a digital camera and analyzed under a research microscope (Zeiss Axioskop with Spot I digital camera, Jena, Germany) with National Institutes of Health (NIH) Image software version 1.61 (Wayne Rasband, NIH, http://rsb.info.nih.gov/nih-image). Plaque areas were adjusted for the cross-sectional vessel cavity area and expressed as a percentage value. All analyses were performed blindly without knowledge of the origin of the samples.

### Leucocytes Migration Assay

Leucocytes were isolated from rabbit blood using RBC lysis buffer. 0.6 × 10^6^ / ml leucocytes used to study chemotaxis in response to various chemokines like MCP-1 (50 ng/ml) and RANTES (100 ng/ml) using 48 well Boyden chemotaxis chamber (Neuro Probe, Inc. Gaithersburg, MD, USA). Lower wells of the chamber were filled with 26uL of chemokines in RPMI 1640 and covered with 5 μm pore size polycarbonate filter membrane. Top wells were filled with 0.6 × 10^6^ / ml leucocytes in RPMI 1640 with 1% FBS. Chemotaxis chamber was incubated for 2 hours at 37°C and 5% CO2 condition. After 2 hourd of incubation polycarbonate membrane was stained with HEMA-3^®^ stain kit (Fisher scientific, Inc. Kalamazoo, MI. USA). Migrated cell were counted at total 200x magnification using Zeiss Microscope, Axiovert 200M. Three high power fields with maximum cell count were counted from each well area.

### siRNA Transfection

Pre-validated Nrf2 and control siRNA were obtained from Invitrogen. Transfection was performed with Lipofectamine 2000 from Invitrogen per manufacturer’s instruction. The efficiency of knockdown and migration assays were performed 72 hours after transfection.

### Statistics

All values in the paper represent mean ± SEM unless otherwise specified. p<0.05 was set as statistically significant differences. All statistics were performed using GraphPad Prism (Version 4.0, GraphPad Software, Inc., La Jolla, CA). For those analyses of dose dependency, a sigmoidal dose-response curve was applied to generate optimized data sets, and meanwhile subjected to a two-way ANNOVA analysis. For bar graphs, data were compared using unpaired, 2-tailed *t* test, one-way ANNOVA, or two-way ANNOVA as specified in figure legends.

## Results

### Treatment with LMDP reduced spontaneous and balloon injury-induced atherosclerotic lesions

**Fig 1:**
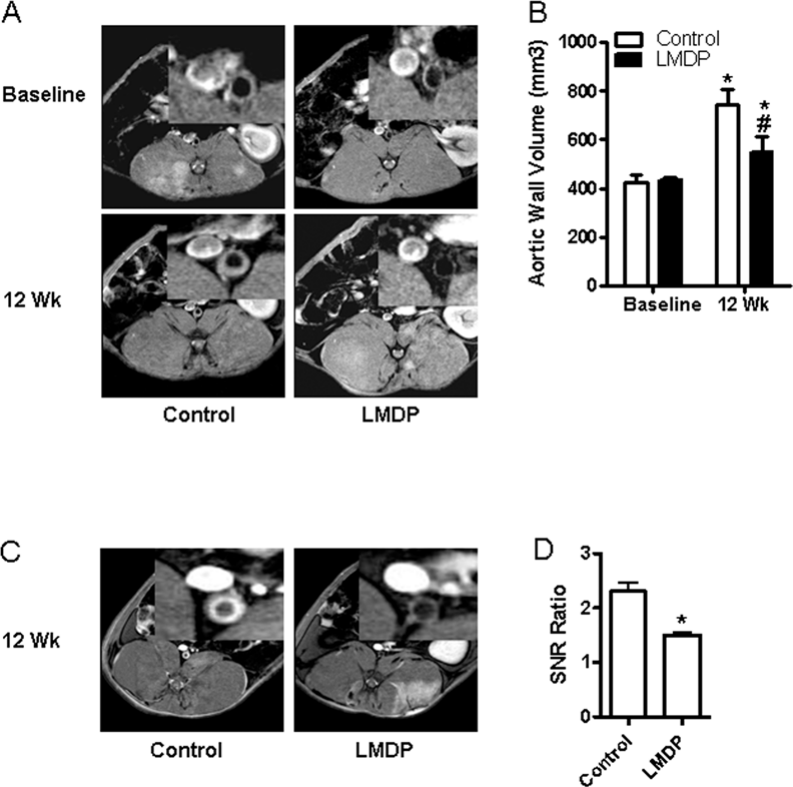
LMDP inhibits balloon injury-induced atherosclerosis. The wall thickness of rabbit abdominal aorta was analyzed by MRI in week one (baseline) or week 12 (12 Wk) after balloon injury. The representative images (A) and quantization data (B) are presented. *p<0.05 vs baseline and ^#^p<0.05 vs control, two way ANOVA, n = 6/group. C and D, Contrast MRI analysis of rabbit abdominal aorta in week 12 after balloon injury. *p<0.05 vs control, student's t-test, n = 6/group.

**Fig 2:**
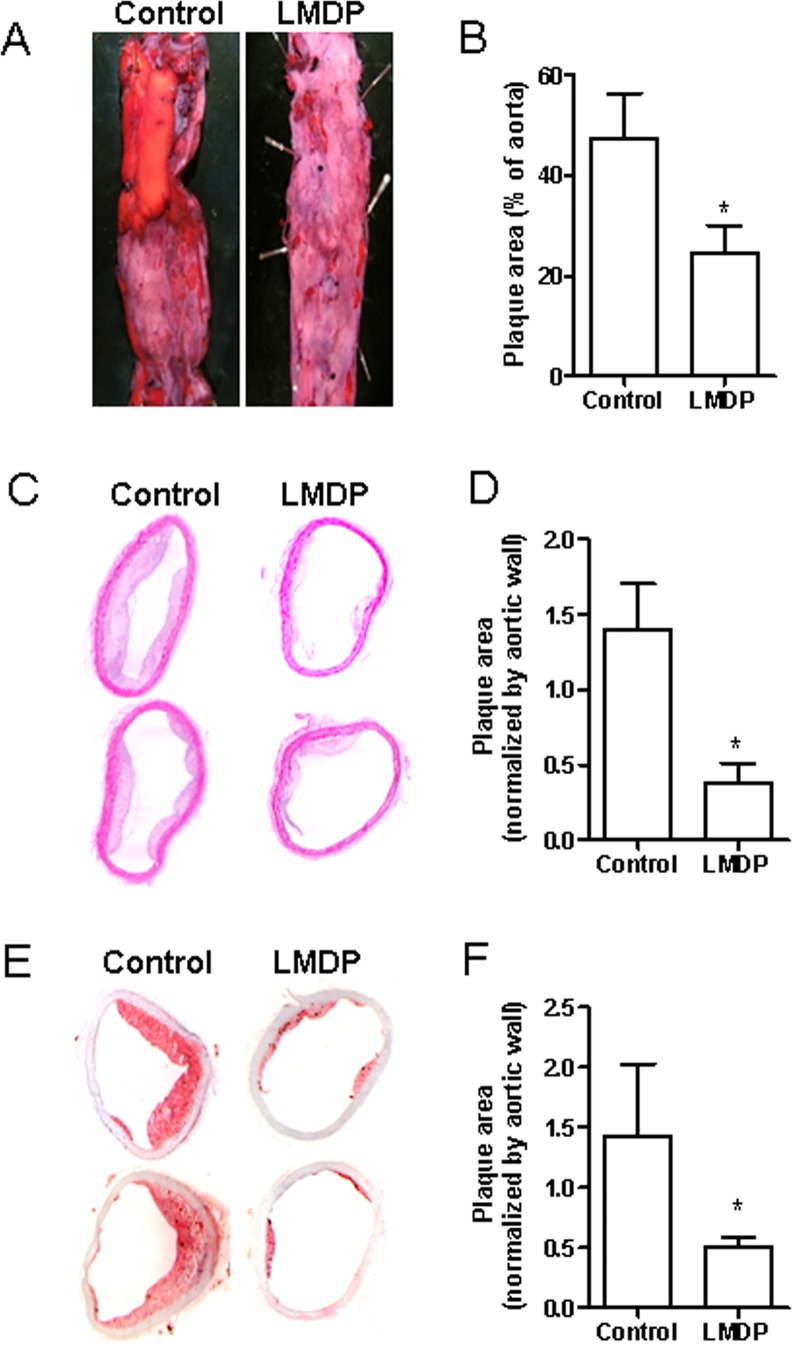
LMDP inhibits both balloon injury-induced and spontaneous atherosclerosis. A, After treatment with LMDP, the rabbit abdominal aorta were collected and atherosclerotic lesions were visualized by oil red o staining. The representative images (A) and quantization data (B) are presented. *p<0.05 vs control, student's t-test, n = 6/group. After treatment with LMDP, the spontaneous atherosclerosis in the thoracic aorta was analyzed by H&E staining (C and D) and oil red o staining (E and F). The representative images (C and E) and quantization data (D and F) are presented. *p<0.05 vs control, student's t-test, n = 6/group.

During the whole duration of treatment with LMDP, no significant differences in food intake and body weight were observed. MRI analyses of abdominal aortic wall thickness, an indicator of balloon injury-induced atherosclerotic burden, show that although no significant difference in abdominal aortic wall thickness between control and LMDP-treated animals was observed one week after balloon-injury (Fig 1A and 1B), significantly reduced aortic wall thickness in LMDP-treated animals was observed 12 weeks after surgery (Fig 1A and 1B). This reduction of abdominal aortic wall thickness in LMDP-treated animals was replicated by contrasting MRI analyses (Fig 1C and 1D). En face analyses confirm that 14 weeks of treatment with LMDP significantly inhibited balloon injury-induced atherosclerotic lesions (Fig 2A and 2B). Notably, 14 weeks of treatment with LMDP also significantly decreased spontaneous atherosclerotic burden in thoracic aorta (Fig 2C–2F).

### Treatment with LMDP did not change plasma lipoprotein profile

**Fig 3:**
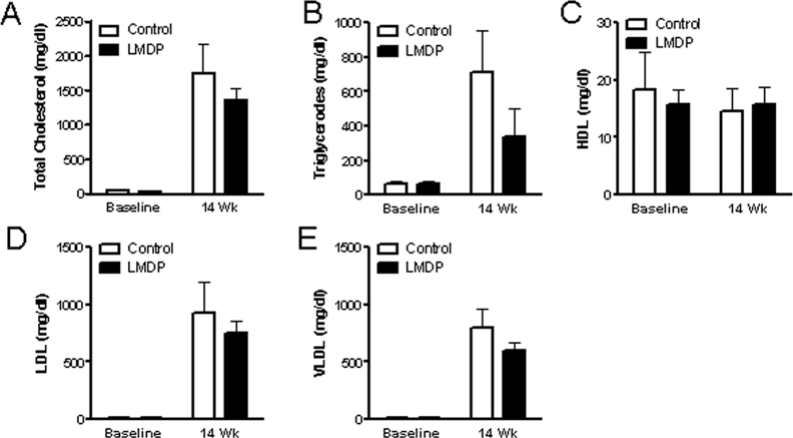
LMDP does not alter lipoproteins. Plasma of rabbits was collected before LMDP treatment (baseline) and after 14 weeks of LMDP treatment (14 Wk), plasma lipoproteins were profiled, and the levels of total cholesterol (A), triglycerodes (B), HDL (C), LDL (D), and VLDL (E) are presented. Two way ANOVA, n = 6/group.

Plasma lipoproteins, in particular LDL and HDL, play a central role in the development of atherosclerosis, and we previously showed that another methylenedioxyphenol derive INV-403 lowers LDL cholesterol through induction of LDL receptor expression [22]. To test if LMDP inhibits atherosclerotic progression through change in lipoprotein levels, plasma of control and LMDP-treated animals were subjected to lipoprotein profiling. Fig 3 demonstrates that after 14 weeks of treatment with LMDP, no significant difference in any fraction of plasma lipoproteins was observed, suggesting that LMDP inhibits atherosclerotic progression through lipoprotein metabolism-independent mechanisms.

### Treatment with LMDP improved vascular function

**Fig 4:**
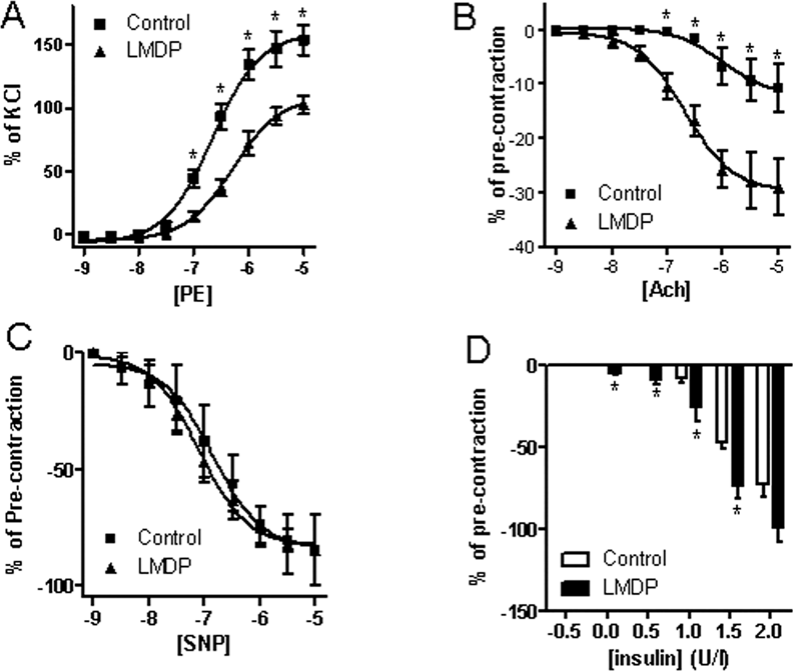
LMDP ameliorates vascular functions. After euthanized, rabbit thoracic aorta were isolated and mounted onto myograph. The aortic responses to phenylephrine (A), acetylcholine (B), sodium nitroprusside (SNP, C), and insulin (D) were analyzed and presented. *p<0.05 vs control, two way ANOVA, n = 6/group.

Vascular dysfunction, characterized by increased sensitivity to vasoconstrictors and decreased sensitivity to vasodilators, is associated with the progression of atherosclerosis. Fig 4A and 4B and Table 1 reveal that 14 weeks of treatment with LMDP significantly reduced the aortic response to phenylephrine (PE, a selective α1-adrenergic receptor agonist that is frequently used as a vasoconstrictor) and enhanced their response to acetylcholine (Ach, a vasodilator primarily through activation of eNOS), supporting that LMDP inhibits atherosclerotic progression through improvement of vascular function. Fig 4C shows that treatment with LMDP did not affect the aortic response to SNP, a NO donor and thus an endothelium-independent vasodilator, suggesting that LMDP primarily target endothelium for its vascular effects. Notably, although treatment with LMDP did not affect the plasma fasting glucose and insulin levels (data not shown), it significantly improved the aortic response to insulin (Fig 4D), another eNOS-dependent vasodilator.

**Table 1:** The EC_50_s and maximal effects of aortic responses.

	Control		Lipoyl-MDP	
	logEC50 (M)	Maximum Effect	logEC50 (M)	Maximum Effect
Ach	-5.99 ± 0.33	-12.27 ± 2.54	-6.67 ± 0.16*	-29.84 ± 2.09*
PE	-6.65 ± 0.08	159.4 ± 6.12	-6.28 ± 0.08	109.1 ± 4.78*
SNP	-6.83 ± 0.19	-84.36 ± 6.24	-7.09 ± 0.16	-83.06 ± 4.95
Insulin	1.72 ± 0.12	-114.7 ± 14.53	1.47 ± 0.139*	-132.5 ± 14.59*

*p<0.05 versus control; Comparison of Fits by Prizm5.

### Treatment with LMDP reduced leukocyte chemotaxis

**Fig 5:**
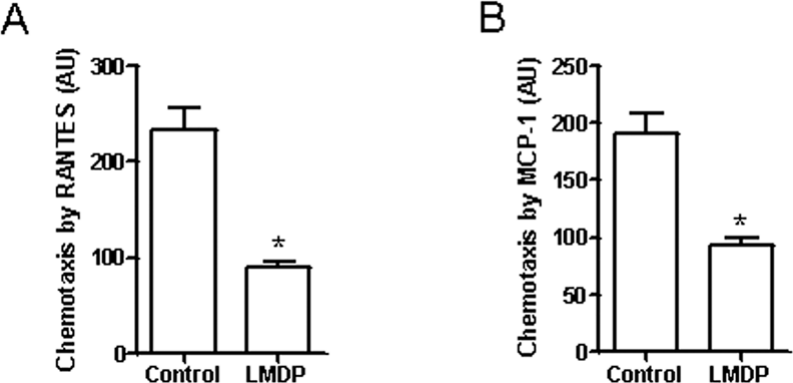
LMDP reduces leukocyte migration. After 14 weeks of LMDP treatment, rabbit leukocytes were isolated and their migratory responses to RANTES (A) and MCP-1 (B) were assessed by Boyden chamber. *p<0.05 vs control, student's t test, n = 6/group.

It comes to be a census that inflammation plays a prominent role in atherosclerosis and its complications, and the chemotaxis of leukocytes is one of the central paradigms of inflammation. To investigate the effect of LMDP treatment on the migration of leukocytes, leukocytes were isolated from the control and LMDP-treated animals, and their chemotaxis in response to RANTES and MCP-1 were assessed with Boyden Chambers. Fig 5 reveals that leukocytes from LMDP-treated rabbits had significantly decreased chemotaxis in response to RANTES and MCP-1.

### LMDP activated cellular Nrf2 signaling

**Fig 6:**
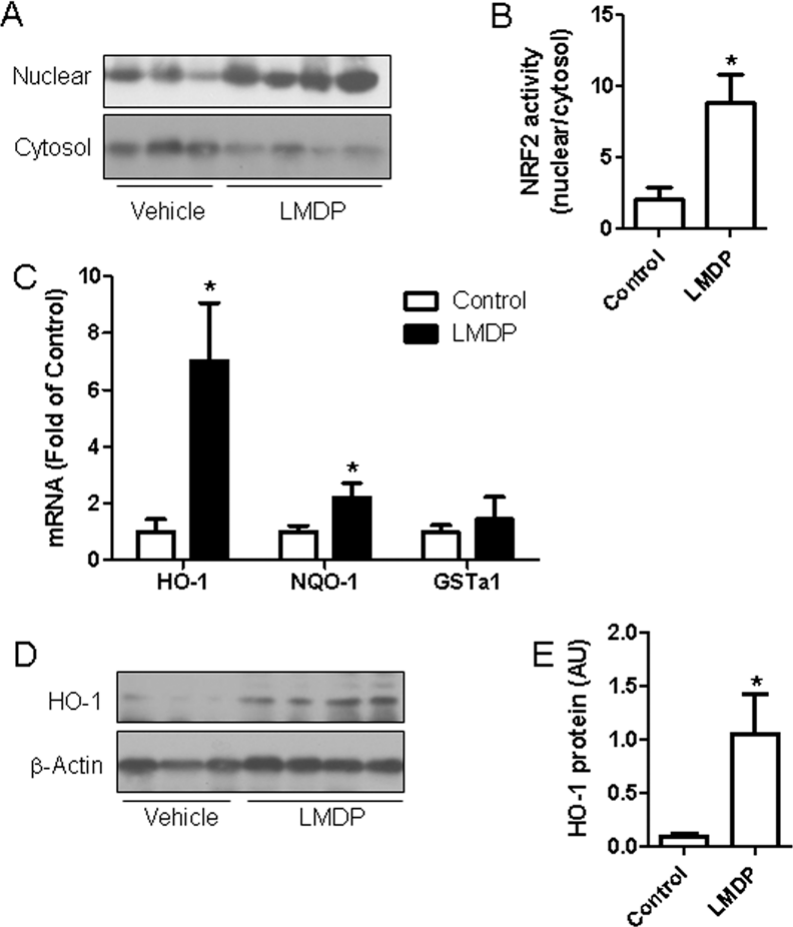
LMDP activates vascular Nrf2 signaling. A, The translocation of Nrf2 in rabbit aorta was assessed by compartmentalization follows by western blot. *p<0.05 vs control, student's t test, n = 5 or 6/group. The representative images (A) and quantization data (B) are presented. C, The mRNA expression levels of the indicated genes in rabbit aorta was analyzed by real-time RT-PCR. *p<0.05 vs control, student's t test, n = 5 or 6/group. D and E, The protein expression of HO-1 in rabbit aorta was analyzed by western blot. *p<0.05 vs control, student's t test, n = 5 or 6/group.

Oxidative stress is one critical component of many inflammatory responses and is implicated in the pathogenesis of vascular dysfunction and atherosclerosis. Nrf2 signaling system is evolved to protect from oxidative stress. Notably, lipoic acid not only has an antioxidant activity but also activates Nrf2 signaling [23,24]. To assess the effect of LMDP treatment on Nrf2 signaling, nuclear and cytosolic proteins were prepared from thoracic aorta and subjected to Nrf2 translocation analysis. Fig 6A and 6B reveal that LMDP treatment significantly increased Nrf2 translocation into nuclear, supporting that LMDP activates Nrf2 *in vivo*. To confirm the activation of Nrf2 signaling by LMDP, the expression of some target genes of Nrf2, including HO-1, NQO-1, and GSTa1 were analyzed by real-time RT-PCR. Fig 6C demonstrates that LMDP significantly increased the mRNA expression of HO-1 and NQO-1, reinforcing that LMDP activates Nrf2 signaling *in vivo*. The induction of HO-1 protein by LMDP treatment was recapitulated by western blot analysis (Fig 6D and 6E).

**Fig 7:**
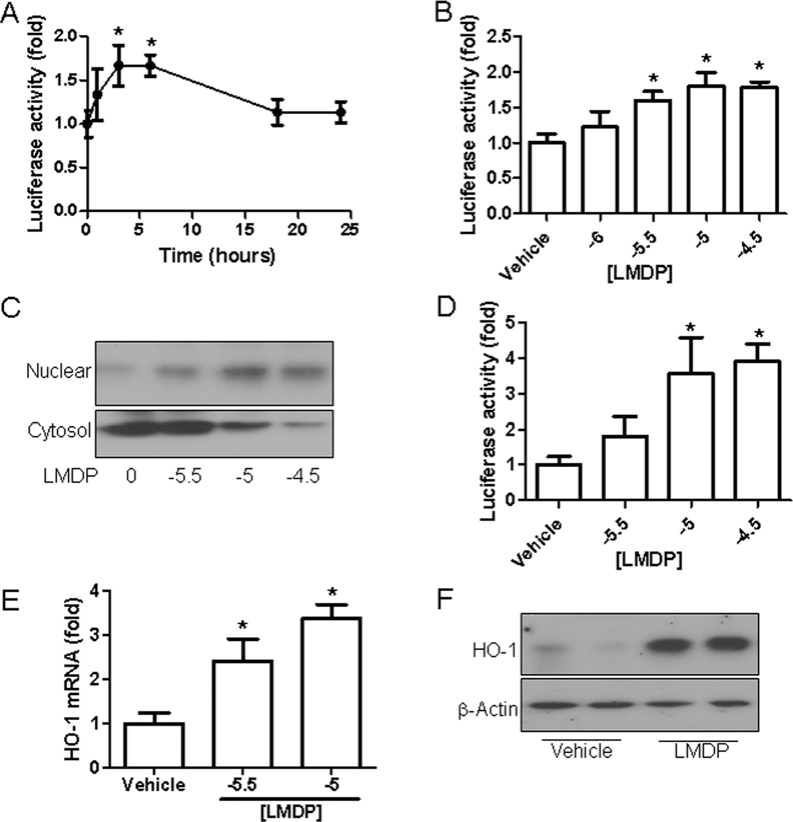
LMDP activates Nrf2 in cultured cells. A and B, COS-7 cells were transfected with Nrf2 reporter construct that express luciferase under the control of Nrf2. After treatment with LMDP (10 μM) for the indicated duration (A) or the indicated concentration of LMDP for 4 hours (B), the luciferase activity was analyzed. *p<0.05 vs 0 hour (A) or vehicle (B), one way ANOVA, n = 5. C and D, mouse abdominal macrophages were treated with the indicated concentration of LMDP for 1 hours, and the translocation of Nrf2 was then analyzed by western blot. *p<0.05 vs vehicle, one way ANOVA, n = 3. E, mouse abdominal macrophages were treated with the indicated concentration of LMDP for 1 hour and the mRNA expression of HO-1 was then analyzed by real-time RT-PCR. *p<0.05 vs vehicle, one way ANOVA, n = 3. F, mouse abdominal macrophages were treated with the indicated concentration of LMDP for 4 hours, and the protein expression of HO-1 was then analyzed by western blot.

To verify the activation of Nrf2 signaling by LMDP, COS-7 cells were used to perform Nrf2 signaling reporter assay. Fig 7A and 7B demonstrate that LMDP time- and dose-dependently increased Nrf2 activity in COS-7 cells, supporting that LMDP may directly regulate Nrf2 signaling. Given that a recent report showed that Nrf2 signaling in bone marrow-derived cells is critical for atherosclerotic progression, we next assessed the effect of LMDP on Nrf2 activation in mouse macrophages. Fig 7C and 7D reveal that LMDP dose-dependently increased Nrf2 translocation. The activation of Nrf2 in mouse macrophages was further confirmed by the increased expression of HO-1 mRNA (Fig 7E) and protein (Fig 7F).

### The reduction of leukocyte chemotaxis by LMDP treatment was mediated by Nrf2 signaling pathway

**Fig 8:**
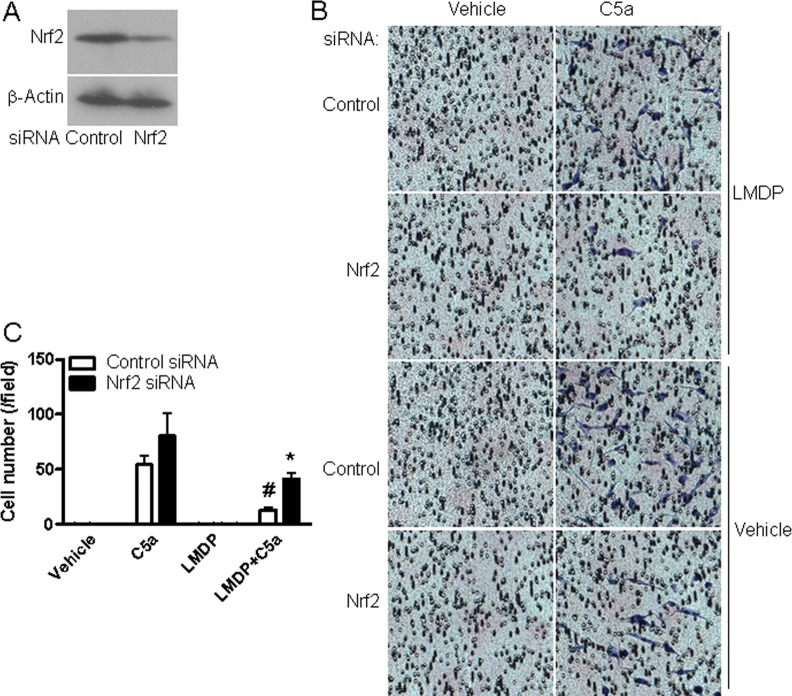
Nrf2 activation mediates the effect of LMDP on macrophage migration. Mouse abdominal macrophages were treated with Nrf2 or control siRNA. The Nrf2 protein expression was analyzed by western blot (A) and the migration in response to C5a in the presence of vehicle or LMDP (B and C) was assessed with Boyden chamber.

Given the marked effect of LMDP treatment on leukocyte chemotaxis and evidence that Nrf2 signaling is implicated in cell migration, we assessed if Nrf2 signaling pathway mediates the effect of LMDP on leukocyte chemotaxis. Fig 8A shows that Nrf2 siRNA decreased the protein expression of Nrf2 in mouse macrophages by about 60% and accordingly reduced the effect of LMDP on macrophage chemotaxis, strongly supporting that Nrf2 activation is implicated in the reduction of leukocyte chemotaxis by LMDP treatment.

## Discussion

Atherosclerosis is one of the most important and common causes of death and disability throughout the world. In the present study, we demonstrate that a new synthesized chemical LMDP markedly inhibits balloon injury-induced and spontaneous atherosclerotic progression in rabbit models, strongly supporting its potential in treatment for human atherosclerosis. LMDP contains the moieties of lipoic acid and methylenedioxyphenol that were shown to inhibit atherosclerotic progression in different animal models [13,16,20]. Notably, while lipoic acid and methylenedioxyphenol were shown to inhibit atherosclerotic progression at least partly through their effects on lipoprotein metabolism [13,22], the present study did not observe any significant effect of LMDP on plasma lipoproteins, suggesting that the anti-atherosclerotic mechanism of LMDP is different from that of lipoic acid and methylenedioxyphenol. Reinforcing this notion, treatment with LMDP did not have a significant effect on body weight, whereas treatment with lipoic acid has consistently been shown to decrease body weight [13,25].

Another important finding of the present study is the demonstration of an important role of Nrf2 signaling pathway in mediating the anti-atherosclerotic effect of LMDP. By far, there is a consensus that atherosclerosis represents a state of heightened oxidative stress in the vascular wall [26], and the Nrf2 pathway is crucial in determining the sensitivity of mammalian cells to oxidative insults through the basal and inducible expression of an abundance of detoxification enzymes, antioxidant proteins, xenobiotic transporters and other stress response proteins [27]. Our data demonstrate that the reduction of atherosclerotic burden by LMDP was paralleled by an increased activation of aortic and monocytic Nrf2 signaling pathway (Fig 6), suggesting that the anti-atherosclerotic effect of LMDP may be mediated by this signaling pathway. Notably, there is still some controversy over the role of Nrf2 signaling in atherosclerotic progression. For example, while whole-body Nrf2 deletion decreases atherosclerosis in ApoE-/- mice [9,10], myeloid deletion of Nrf2 increases atherosclerosis in LDLR^-/-^ mice [11]. The pro-atherosclerotic effect of Nrf2 shown in whole body deficient mice was primarily attributed to altered liver and plasma cholesterol and cholesterol crystal-induced inflammasome activation [9,10]. Our data show that the anti-atherosclerotic effect of LMDP was coincident with a lack of any significant effects on plasma lipoproteins. This is consistent with the above notion, and strongly supports that it is possible to pharmacologically differentiate the anti-atherosclerotic function of Nrf2 from its regulation on lipid metabolism.

Nrf2 has many target genes that can exert different functions of Nrf2. Interestingly, our data reveal that not all target genes of Nrf2 are equally affected by LMDP treatment (Fig 6C), providing a potential interpretation for the aforementioned discrepancy between phenotypes of knockout mice and results of pharmacological studies: The complete deficiency of Nrf2 in knockout mice makes their phenotypes reflect all functions of Nrf2, whereas the differential regulation of Nrf2 target genes by pharmacological activation makes its effects reflect only partial functions of Nrf2. This is consistent with a most recent study demonstrating that CD36, one of the target genes of Nrf2 determines the effects of Nrf2 on atherosclerosis through unknown mechanism [28]. To address whether LMDP activates anti-atherosclerotic genes but not those of lipid metabolism, profiling the expression of Nrf2 target genes in response to LMDP treatment is undergoing.

Vascular function, in particular endothelial dysfunction characterized by a decreased vasodilator response to acetylcholine, is strongly correlated to the progression of atherosclerosis in humans and animal models. Interestingly, our data show that treatment with LMDP markedly improved the aortic response to acetylcholine and insulin, suggesting that the anti-atherosclerotic effects of LMDP may be mediated by the amelioration of endothelial function. This is consistent with studies showing that the anti-atherosclerotic effect of lipoic acid is paralleled by an improvement in endothelial function [16]. However, LMDP also reduces the aortic contractile response to phenylephrine, whereas lipoic acid does not affect aortic contractility [16], suggesting that the effect of LMDP on vascular function may also be unique. Oxidative stress has been shown to play a critical role in vascular function regulation, and Nrf2 signaling pathway is one of the most important cellular antioxidant components. More importantly, HO-1, one critical target gene of Nrf2, was shown to moderates the contractility of vascular smooth muscle through nitric oxide-independent mechanism [29]. Our data demonstrate that treatment with LMDP not only activated Nrf2 (Fig 6A and 6B), but also markedly increased HO-1 expression, suggesting that the vascular effects of LMDP may also be mediated by Nrf2/HO-1 pathway.

The recruitment of monocytes into vascular wall and their subsequent development to foam cells are central in the pathophysiology of atherosclerosis [30]. The improvement of vascular function is believed to inhibit adhesion of monocytes and thus reduce their recruitment into vascular wall. Notably, our data demonstrate that LMDP treatment also markedly reduced the chemotaxis of isolated leukocytes, suggesting that inhibition of monocyte/macrophage recruitment may be the critical target for its anti-atherosclerotic effect. This is somehow consistent with studies showing that lipoic acid inhibits leukocyte migration into the central nervous system in the mouse model of acute experimental allergic encephalomyelitis through unknown mechanisms [31]. Notably, our data indicate that the inhibition of monocyte chemotaxis by LMDP may also be Nrf2 activation-dependent, as knock-down of Nrf2 significantly reduced the effect of LMDP on macrophage chemotaxis. This is consistent with a recent report showing that Nrf2^-/-^ macrophages have increased migration in response to MCP-1 [11].

In conclusion, our data demonstrate that LMDP inhibits atherosclerotic progression in a model of atherosclerosis, which may be mediated by activation of Nrf2 signaling and subsequent inhibition of recruitment of monocytes/macrophages. These findings may have implications for further testing of this novel compound in the treatment of atherosclerosis.

## Supporting Information

S1 Appendix Letter from Dr. Rajagopal Desikan showing the source of LMDP. (PDF)
