# Supplementary material for: Lipoicmethylenedioxyphenol Reduces Experimental Atherosclerosis through Activation of Nrf2 Signaling
Source: PLoS One. 2016 Feb 9;11(2):e0148305. doi: 10.1371/journal.pone.0148305 (PMC4747573; doi:10.1371/journal.pone.0148305)
Supplement: S1 Appendix — (PDF) [file pone.0148305.s001.pdf]

VIT®
UNIVERSITY
(Estd. u/s 3 of UGC Act 1956)
Vellore - 632 014, Tamil Nadu, India

10th December 2015

TO WHOM SO EVER IT MAY CONCERN

I hereby wish to state that the chemical compound, LA-MDP, mentioned in the manuscript titled "Lipoicmethylenedioxyphenol Reduces Experimental Atherosclerosis through Activation of Nrf2 Signaling" was synthesized by me when I was working for InVasc Therapeutics, Columbus, Ohio, 43210. If you have any question or concern regarding this, please do not hesitate to contact me either through email or contact phone number.

Thank you,

Sincerely yours,

Dr. Rajagopal, Desikan, Ph.D.
Professor,
Dept. of Chemistry and Pharmaceutical chemistry
School of Advanced Science
VIT University
Vellore, 632014. Tamil Nadu, India
Phone : 9578935164
Email : rajagopal.desikan@vit.ac.in
